# Incentive effect of tax preferences towards the technological innovation of enterprises——Based on China’s GEM listed companies

**DOI:** 10.1371/journal.pone.0282692

**Published:** 2023-04-14

**Authors:** Liang Ding, Yunfeng Wu, Junxia Long

**Affiliations:** 1 School of International Trade and Economics, Jiangxi University of Finance and Economics, Nanchang, China; 2 School of Public Finance and Public Administration, Jiangxi University of Finance and Economics, Nanchang, China; University of Salento, ITALY

## Abstract

The long R&D process, the high risk, and the externalities of technological innovation are challenges that enterprises have to meet when making decisions on R&D investment. Governments share this risk with enterprises through preferential tax policies. We summarized China’s preferential tax policies related to enterprises and R&D innovation, and used panel data of listed enterprises from 2013 to 2018 in the Growth Enterprises Market (GEM) of the Shenzhen Stock Exchange to explore the incentive effects of current tax policies on the R&D innovation of enterprises. Through empirical analysis, we found that tax incentives significantly motivate R&D innovation input and promote output. In addition, we found that the income tax incentives are greater than that of the circulation tax, since the profitability of enterprise has a positive correlation with R&D investment. Meanwhile, the size of the enterprise is negatively correlated with the intensity of R&D investment.

## I. Introduction

The uncertainty of R&D restricts an enterprise’s R&D investment. For enterprises, R&D investment is a high-risk investment, and some enterprises are reluctant to invest in R&D due to their risk avoidance considerations. Preferential tax policies could reduce the risk of enterprise R&D investment. Tax incentives are essential for the government and enterprises in order for them both to share risk together. Preferential tax policies include tax credits, tax exemptions, and tax refunds, among others. The risk incurred by enterprises changes depending on the size of government compensation.

Different preferential policies have pros and cons. For example, using fiscal subsidies has clear policy goals. However, the implementation of the policy has a time lag. It is also difficult to reach and benefit all enterprises. At the same time, fiscal subsidies will have an alternative effect on R&D activities. The preferential tax policies will play a role through the power of the market and will target a wider range of enterprises. In practice, the preferential tax policy is the main means for most countries to encourage scientific and technological innovation around the world.

Researchers have found evidence of the effectiveness of preferential tax policies on enterprise R&D in many countries. For OECD countries, Bloom et al. [1] analyzed the data from nine OECD countries in the 1980s and 1990s, and found that tax incentives increased the intensity of R&D investment. McKenzie and Sershun [2] used a dynamic fixed effect model, and found that both direct and indirect tax preference methods have a significant impact on enterprise R&D expenditure. Further, the long-term effect is even more significant based on 19 years of panel data from 9 countries. Koga [3] used panel data from 904 Japanese industrial enterprises from 1989 to 1998, and found that tax incentives had an incentive effect on enterprise R&D investment. Czarnitzki et al. [4] analyzed the impact of the R&D tax credit on Canadian corporate innovation activities, and found that companies receiving the tax credit had more product innovation, as well as sales growth of new and improved products. However, the tax credit did not demonstrate significant improvement in corporate performance. Doh and Kim [5] explored the impact of government tax incentives on innovation for small and medium-sized Korean enterprises (SMEs). The results show that government tax incentives positively affect industrial innovation.

According to Herrera and Sanchez-Gonzalez [6], although tax incentives increase corporate innovation, the effect depends on the size of the business. Enterprises with different sizes show different responses to preferential tax policies. Compared with small enterprises, the incentive effect on large enterprises is more clear. Thus, US tax incentives create a more obvious incentive for corporate R&D spending. Hanel [7] found that large Canadian companies were more frequently using preferential R&D tax deductions, and that it was easier for large companies to apply for tax deductions. Koga [3], taking the value of large and medium-sized Japanese manufacturing enterprises as the sample, found that the calculated R&D tax elasticity number of large and medium-sized manufacturing enterprises is -1.036 and -0.118 respectively, and that tax incentives mainly encourage the R&D of large enterprises. Ghazinoory and Hashemi [8] compared the use of both incentives by Iranian high-tech SMEs and large firms through a factorial design technique. Results showed that for SMEs, tax exemption has a significant effect on R&D investment. Kobayashi [9] used Japanese SME data to show that the effectiveness of tax incentives on technological innovation is related to the scale of the enterprise, the industry, and the liquidity of capital available. Taking into account the specific characteristics of small and medium-sized enterprises, Castellacci and MeeLie [10] found that tax incentives on technological innovation have strong effects on the service sector and weak effects on the lower-tech industries. Dai and Chapman [11] found evidence that tax incentives have stronger innovation impact on firms with greater programming experience and innovation experience.

In terms of preferential tax policies in China, Huang et al. [12] believe that China’s preferential tax policies for high-tech enterprises are mainly by directly preferential policies, while indirect preferential policies on the accelerated depreciation, the additional deduction of R&D expenses, the accelerated depreciation of fixed assets, and R&D fund reserves are lower. Wan et al. [13] used the SAR Tobit model and panel data from China’s 30 regional hi-tech industries from 2004 to 2016, and found that the preferential tax policy has an obvious crowding effect on R&D technical efficiency and business transformation efficiency. Sun et al. [14] analyzed the implementation of tax incentives for high-tech enterprises in Shanghai, Guangdong, Zhejiang, and Jiangsu. It was found that low tax incentives had a significant incentive effect on R&D investment of high-tech enterprises. As found was what, tax incentives strongly supported independent innovation and industrial transformation. Qin and Huang [15] studied Chinese high-tech listed companies from 2007 to 2019. They argued that the higher the proportion of government subsidies for high-tech enterprises, the more positive effects of tax incentives on non-inventive innovation efficiency. However, it does not play a significant role in promoting inventive innovation efficiency.

In the comparison of the incentive effect of value-added tax (VAT) preference and corporate income tax preference, Kuang and Xiao [16], using OLS regression method, found that a 1% lower corporate income tax burden will increase the R&D expenditure by 0.99%. However, a 1% lower circulation tax can only increase the R&D expenditure by 0.4%. Therefore, compared with the circulation tax, the incentive effects of an income tax are more significant. Sun et al. [17] studied the effect of VAT incentives on listed companies of new energy industry through the Difference-In-Difference (DID) approach. The results showed that VAT refunds for new energy industry have a time lag impact, and the effects vary considerably over time seen by examining the dynamic effects of VAT incentives.

On the impact of an enterprise income tax on enterprise technology innovation, Li et al. [18] used investment intensity as the explained variable, with enterprise income tax preferential as the explanatory variable. They found that tax preferential policies have a positive effect on R&D investment intensity. For an income tax, the preferential rate increases every 1%, leading state-owned enterprises and non-state-owned enterprises to increase R&D investment intensity 0.0944%, and 0.0218% respectively. Yang et al. [19] conducted an empirical analysis using panel data of listed companies on the GEM, and the results show that for each enterprise income tax discount increase of 1%, the R&D investment will increase by 0.202%. This demonstrates that the enterprise income tax discount has a significant positive effect on increasing innovation investment. Li et al. [20] used panel data of China’s listed private enterprises from 2009 to 2013, and found that corporate income tax incentives to a certain extent improved the innovation performance of enterprises, and innovation input played a mediating role. However, the preferential income tax policies for high-tech enterprises may be used to evade paying taxes by some enterprises.

Throughout the previous literature review, we found that scholars have conducted substantial research in the field of tax preferential policies in order to promote enterprise research and innovation. The conclusions are very fruitful and have great reference value. However, there are still some limitations to the research. Firstly, the most researched target is corporate income tax, and the incentive effect of R&D innovation is rare. Secondly, the research is mainly from a macro perspective, and few researchers have focused on the mechanisms regarding tax incentives on R&D investment. Our paper targets the listed enterprises on the Shenzhen Stock Exchange. It investigates the impact of preferential tax policies on enterprise R&D in China. Through empirical analysis of the relevant data, we calculate the intensity of enterprise R&D innovation influenced by the preferential tax policies under China’s current tax environment. We evaluate the implementation effect of preferential tax policies of different taxes on R&D investment. Through empirical analysis, we found that from an overall perspective, income tax incentives significantly motivate R&D innovation input and promote output. The income tax incentives have greater effects than the circulation tax. The enterprise profitability and enterprise R&D investment have a positive correlation, and the enterprise size has a negative correlation with R&D intensity.

The remainder of the paper is structured as follows; Section II summarizes the recent preferential tax policies on R&D in China. In Section III, we discuss our hypothesis and model. Section IV holds description of the data and empirical results. Section V is our conclusion and policy suggestions.

## II. Preferential tax policies on R&D in China

Since the 1990’s, China has introduced many preferential tax policies in order to encourage enterprise technology innovation. Up to now, China’s preferential tax policy system that encourages enterprise innovation activities involves almost all types of taxes. Most policies are concentrated in the fields of enterprise income tax, personal income tax, and VAT. We summarized the preferential tax policies on VAT and income tax for the most recent years in Tables 1 and 2 respectively.

**Table 1:** Preferential VAT policies.

Document	Main Contents
Taxation [2011] No.100	For the average taxpayer selling software products that are produced by themselves, after being taxed according to the statutory tax rate, the portion of the actual tax above 3% will be refunded.
Taxation [2016] No.121	If the R&D institution purchases domestic equipment for R&D of new technologies and new products, the VAT can be refunded in full.
Taxation [2016] No.121	Imported instruments and equipment directly used for scientific research, scientific testing, and teaching shall be exempted from VAT.
Taxation [2016] No.36	Taxpayers providing technology transfer, technology development, and related technical consultation and technical services shall be exempted from VAT.

**Table 2:** Income tax policies.

	Document	Main Content
Business Income Taxes	The Enterprise Income Tax Law of the People’s Republic of China, Article 28, Paragraph 2	High-tech enterprises mainly supported by the state shall collect enterprise income tax at a reduced rate of 15%.
	The Implementation of the Enterprise Income Tax Law of the People’s Republic of China, Article 42	The expenses incurred by the enterprise on the education of employees, if not exceeding 8% of the total salary, shall be allowed to be deducted from the enterprise income tax. The excess part shall be carried forward and deducted in the later tax years.
	Taxation [2018] No.76	From January 1, 2018, enterprises with the qualification of high-tech enterprises, or small and medium-sized enterprises whose outstanding losses incurred in the previous five years, shall be allowed to make up for the following year, and the maximum carry-forward period shall be extended from 5 years to 10 years.
	Taxation [2019] No.66	The fixed assets of all manufacturing and information transmission software, and information technology services, shall be shorten the depreciation time. The new research and development technology and equipment purchased by small and low-profit enterprises are below million yuan, can be deducted at one time before tax.
	Taxation [2017] No.34	If the R & D expenses actually incurred in R & D activities, and do not form the intangible assets included in the current profit and loss, they shall be deducted at 75% from January 1, 2018 to December 31, 2020. The intangible assets shall be amortized at 175% of the intangible assets cost during the above period.
	Taxation [2018] No. 99	
Individual Income Tax	Taxation [2018] No.58	The cash rewards given to scientific and technological personnel by non-profit research and development institutions and institutions of higher learning from the transformation income of their post scientific and technological achievements, may be reduced by 50% into the "salary and salary income" of scientific and technological personnel in the current month when paying individual income taxes.
	Taxation [2016] No.101	Equity rewards given by high-tech enterprises shall allow individuals to pay personal income tax until the time of obtaining dividends or the transfer of equity. When small and medium-sized high-tech enterprises convert individual shareholders capital into capital stock with retained income and capital reserve, they may pay individual income tax in installments within five years according to the actual circumstances.

In sum, the government has made great efforts to enact preferential tax policies in order to encourage enterprises to actively increase R&D research. Meanwhile, the current challenge is that a comprehensive policy framework has not been established. For example, many preferential policies have simply been in the form of Notice and Method, scattered in the tax law, or taken place in the absence of a system, none of which has formed a unified legal system. In the process of the implementation of the preferential tax policy, it is a lack of proper cohesion and a lack of transparency that reduces the tax preferential effect. The preferential tax policies to encourage enterprises’ scientific and technological innovation are mainly based on income tax, that is not consistent with a structural system based on circulation tax. Income tax preferential is related to enterprise profits. This means that only profitable enterprises can enjoy preferential policies. Enterprises in the early stage of entrepreneurship cannot enjoy tax incentives due to possible small profits or losses. The current incentive policies on technology innovation tax are concentrated downstream within the industrial chain, paying close attention to the results rather than the process. Only successful R&D enterprises can enjoy preferential policies, prohibiting some enterprises from taking advantage of these preferential policies until they mature.

## III. Hypothesis and model

The technological innovation of enterprises can be divided into two stages. The early stage is mainly about the input of R&D, and the later stage is about technology innovation output. However, technological innovation is not achieved overnight. The long R&D process, the high risk, and the externalities of technological innovation are all problems that challenge enterprises. These challenges weaken the enthusiasm for an enterprise’s technological innovation. As a means of national macro adjustment, preferential tax policies can reduce the risk of an enterprise’s technological investment in innovation, thus reducing the nominal capital investment in R&D and improving the profitability of the enterprise through innovation.

Many scholars have proven through a large and significant number of studies that preferential tax policies have a positive impact on technological innovation. Therefore, we propose the first hypothesis:

H1: Preferential tax policies promote technology-based enterprises and are intended to increase investment in R&D.

In addition, the reduction in taxes for these enterprises will bring extra profit and increase enterprises’ competitiveness, so that enterprises have more funds that they can possibly invest in R&D and innovation. In terms of measuring the achievements of innovation of enterprises, the existing research mostly starts from the intangible output of innovation, then establishes the evaluation indicators represented by patent rights. For example, Cloodt et al. [21] select the number of patent authorizations obtained by the enterprise as the output index. We use the number of patent rights and other forms of intellectual property rights of a business to represent the level of an enterprise’s technological innovation. We, therefore, propose a second hypothesis:

H2: Preferential tax policies can promote the technological innovation output of enterprises.

A tax deduction on different types of taxes may have different impacts on the enterprise’s R&D activities. We have an interest in identifying the effects of tax preferential policy on various types of taxes. The third hypothesis is presented:

H3: Preferential income tax has more benefits than preferential circulation tax in encouraging enterprises to carry out R&D activities, thus increasing R&D investment and improving the output of innovation.

In order to explore the impact of income tax incentives on the investment of enterprise R&D, referring to the model of Zhou et al. [22], we will use the following model (Model 1):

$${\mathrm{RD}}_{\mathrm{it}}=\alpha_{0}+\alpha_{1}{\mathrm{TS}}_{\mathrm{it}}+\alpha_{2}{\mathrm{TTAX}}_{\mathrm{it}}+\alpha_{3}{\mathrm{ROE}}_{\mathrm{it}}+\alpha_{4}{\mathrm{SIZE}}_{\mathrm{it}}+\alpha_{5}{\mathrm{SM}}_{\mathrm{it}}+\alpha_{6}{\mathrm{LEV}}_{\mathrm{it}}+v_{i}+\varepsilon_{\mathrm{it}}$$ (1)

In the Model 1, RD is R&D investment; TS is Corporate income tax incentives; TTAX is the circulation tax; ROE is the return on equity; SIZE is enterprise size; SM is Net operating profit rate; LEV is asset-liability ratio; α_0_ is a fixed intercept term; v_i_ represents the individual effect of each enterprise R&D investment; and ε_it_ represents the random perturbation term that changes with the enterprise and year.

In order to explore the impact of income tax incentives on innovation output, referring to the model of Li et al. [20], we will use the following model (Model 2):

$${\mathrm{effect}}_{\mathrm{it}}=\alpha_{0}+\alpha_{1}{\mathrm{TS}}_{\mathrm{it}}+\alpha_{2}{\mathrm{TTAX}}_{\mathrm{it}}+\alpha_{3}{\mathrm{ROE}}_{\mathrm{it}}+\alpha_{4}{\mathrm{SIZE}}_{\mathrm{it}}+\alpha_{5}{\mathrm{SM}}_{\mathrm{it}}+\alpha_{6}{\mathrm{LEV}}_{\mathrm{it}}+v_{i}+\varepsilon_{\mathrm{it}}$$ (2)

The effect is innovative output performance; other independent variables are the same as in model 1; α_0_ is a fixed intercept term, and v_i_ represents the individual effect of the innovative output performance of each enterprise, ε_it_ represents the random perturbation term that changes with the enterprise and year.

## IV. Data and test

### 4.1. Data description

We use the listed enterprises from the Growth Enterprises Market (GEM) of the Shenzhen Stock Exchange as our sample since the GEM-listed companies are characterized by high technology with high rates of growth. In general, those companies use new technology, new materials, new services, new energy sources, and new agricultural methods. Compared to other enterprises, they pay more attention to R&D, have more innovation strength, and are more sensitive to government implementation of tax preferential policies. We selected GEM panel data from 2013 to 2018, and excluded the companies which did not disclose their R&D investment, companies with an actual tax rate or adjusted taxable income that is less than 0, companies with missing or abnormal main explanatory variables, as well as the specially treated companies. All the data and information are collected from the CSMAR database.

The investment in R&D (RD) is the basis for enterprise innovation activities. The scale of enterprise R&D investment is measured by the ratio of R&D expenditure to operating income. Innovative output (effect) is measured by the ratio of capitalized R&D expenditure to net profit. Innovative patent (patent) uses the natural log of the number of patent applications. Corporate income tax incentives (TS) are measured by the basic income tax rate (25%) minus the ratio of actual tax expenses to profit.

As to the circulation tax (TTAX), in previous studies, the circulation tax burden is studied using two methods to get either positive or negative indicators. Some scholars select the 17% tax rate minus the tax and additional percentage of operating income as the positive index to measure the preferential tax rate of the circulation tax, that is, the actual circulation tax rate. However, considering that the corporate VAT rate is a progressive rate, the above methods may result in a large deviation in the measurement of the corporate circulation tax. According to Tong et al. [23], they recalculate the circulation tax burden through the urban construction maintenance tax, by using the ratio of urban maintenance and construction taxed on income divided by 7%. However, the urban maintenance and construction tax also has a progressive tax rate, so this paper adopts the same method to calculate the circulation tax by the education surcharge tax. Therefore, TTAX is a reverse indicator, which means the greater the tax burden of the circulation tax, the smaller the circulation tax the preferential enterprises will have.

We choose the following factors as control variables: enterprise size (SIZE); asset-liability ratio (LEV); Return on Equity (ROE); Net operating profit rate (SM). A descriptive statistical analysis of the mean, maximum, minimum, and variance of various variables is in Table 3.

**Table 3:** Descriptive statistics.

Variable	Obs	Mean	Std. Dev.	Min	Max
RD	2,420	7.19907	6.082463	0.02	72.75
TS	2,420	13.80765	4.621666	0.008555	24.99958
TTAX	2,420	6.634345	4.324178	0	27.22415
RDRP	1,771	25.2711	76.77733	0.55	3182
effect	2,420	7.898884	57.72032	-530.93	2259.96
patent	519	3.144422	1.105229	0.6931472	5.823046
LEV	2,420	29.05537	16.02809	0	81.17005
ROE	2,420	8.130443	5.761979	0.1208	48.9547
SIZE	2,406	21.20972	0.803645	18.96066	24.76087
SM	2,420	14.56195	10.08231	-107.441	120.8916

Of all the 2,420 samples, the R&D investment ratio varies from 0.02% to 72.75%, and the gap is relatively obvious. The average R&D investment ratio is 7.20%. The maximum value of TS in the sample is 24.99%, indicating that the actual tax burden rate of the enterprise is close to 0. The mean of TS is 13.81% with a standard deviation of 4.62, indicating that the overall sample difference is not very large. The average of circulation tax burden (TTAX) is 6.63%. The minimum is 0 and the maximum is 27.22%. The wide range of TTAX indicates that the circulation tax incentives vary across firms. The average Innovative output performance (effect) is 7.90% with a standard deviation of 57.72. This indicates that the gap between innovation output is relatively large. Innovative patent (patent), which is the natural log of patent applications, ranges from 0.693 to 5.823, with an std of 1.105. This indicates a certain gap between different enterprises.

The average Asset-liability ratio (LEV) is 29.06%, the minimum is 0 and the maximum is 81.17%. The selected sample is mainly small and medium-sized listed enterprises that generally choose bond financing so the risk is relatively high and the asset-liability ratio is relatively large. The large difference in ROE is caused by the different industries in the sample. In general, ROE in the industries of agriculture, forestry, livestock, fisheries, and construction industries is relatively low. While at the same time, being relatively high in information transmission, software, and the information technology service industries. The enterprise size (SIZE) ranges from 18.96 to 24.76, indicating that the gap between enterprises is relatively small, while the Net operating profit rate (SM) varies widely across enterprises.

### 4.2. Regression analysis

We used regression analysis of the panel data from listed the GEM from 2013 to 2018, using Model 1 to test the impact of income tax incentives on the investment of enterprise R&D. The results are shown in Table 4.

**Table 4:** Tax incentives on R&D.

Variable	(1)	(2)	(3)	(4)	(5)
	RD	RD	RD	RD	RD
TS	0.348***	0.344***	0.344***	0.357***	0.369***
	(0.0230)	(0.0220)	(0.0221)	(0.0221)	(0.0218)
TTAX	0.281***	0.182***	0.181***	0.199***	0.143***
	(0.0249)	(0.0248)	(0.0251)	(0.0252)	(0.0254)
LEV		-0.0964***	-0.0966***	-0.0865***	-0.0443***
		(0.00661)	(0.00663)	(0.00734)	(0.00851)
ROE			0.00435	0.00537	-0.112***
			(0.0191)	(0.0191)	(0.0225)
SIZE				-0.248*	-0.652***
				(0.144)	(0.148)
SM					0.143***
					(0.0153)
constant	0.446	3.958***	3.927***	8.538***	14.95***
	(0.378)	(0.435)	(0.456)	(2.965)	(2.993)
Observations	2,420	2,420	2,420	2,406	2,406
R-squared	0.125	0.196	0.196	0.201	0.230
F	172.49***	195.94***	146.91***	121.07***	119.1***

Note:

***、**、* are significant at 1%, 5%, and 10% respectively; the coefficient t is in parentheses.

The coefficient of TS is 0.369 and is significant at 1%, indicating that an income tax discount has a significant positive impact on the intensity of R&D investment. The increase of an income tax discount will increase enterprise investment in R&D. When income tax is deducted by 1%, the enterprise R&D investment increases by 0.369%. Getting more income tax incentives can improve the intensity of enterprise R&D investment, which is consistent with the empirical research results of Wang [24]. The coefficient of TATX is 0.143 and is significant at the level of 1%, indicating that the circulation tax is positively related to R&D investment, and the preferential circulation tax will inhibit enterprise R&D investment. SM is positively and significantly related to R&D investment, which demonstrates that the profitability of enterprises has a positive correlation with R&D investment. SIZE and LEV are negatively and significantly related to R&D investment, which demonstrates that the size and asset-liability ratio of enterprises are negatively correlated with the intensity of R&D investment.

In model 2, we explore the impact of income tax preference and circulation tax preference on the performance of enterprise R&D output. The results are shown in Table 5.

**Table 5:** Tax incentives on R&D output performance.

Variable	(1)	(2)	(3)	(4)	(5)
	effect	effect	effect	effect	effect
TS	0.340***	0.340***	0.299***	0.278***	0.258***
	(0.0768)	(0.0768)	(0.0762)	(0.0775)	(0.0772)
TTAX	0.0421	0.0510	0.150*	0.130	0.230**
	(0.0832)	(0.0865)	(0.0867)	(0.0880)	(0.0902)
LEV		0.00866	0.0205	-0.00212	-0.0778***
		(0.0231)	(0.0229)	(0.0257)	(0.0301)
ROE			-0.474***	-0.462***	-0.253***
			(0.0658)	(0.0667)	(0.0798)
SIZE				0.847*	1.572***
				(0.502)	(0.523)
SM					-0.256***
					(0.0541)
Constant	0.952	0.637	4.016**	-12.93	-24.42**
	(1.262)	(1.516)	(1.572)	(10.37)	(10.61)
Observations	2,420	2,420	2,420	2,406	2,406
R-squared	0.008	0.008	0.029	0.030	0.039
F	9.86***	6.62***	18.02***	14.88***	16.24***

Note:

***、**、*are significant at 1%, 5%, and 10% respectively; the coefficient t is in parentheses.

In Table 5, the coefficient of TS is 0.258 and is significant at 1%, indicating that income tax preference has a significant positive impact on the performance of R&D output. Increasing the income tax preference margin will increase the performance of enterprise R&D output. Increasing the income tax preference by 1%, the average performance of R&D output increases by 0. 258%. Enterprises getting more income tax benefits can improve the performance of R&D and enterprise output. Before adding the control variable, the TTAX is not significant at the level of 5%. After adding the SM, the TTAX will inhibit the R&D and innovation output of enterprises to a certain extent.

### 4.3. Robustness test

The R&D investment could be either financial input or human capital input. The size of the tax burden will have an impact on the human capital input to R&D. Enterprises can deduct pre-tax profits for qualified training costs and benefits of specific research and development personnel. This will reduce the operating costs of enterprises, and then promote innovation investment. We use the proportion of R&D personnel to total employees (RDRP) to replace the RD in the original model to measure the intensity of enterprise R&D investment as a robust test of the original model. The regression analysis results are shown in Table 6.

**Table 6:** Robustness tests of tax incentives on R&D.

Variable	(1)	(2)	(3)	(4)	(5)
	RDRP	RDRP	RDRP	RDRP	RDRP
TS	0.824***	0.812***	0.823***	0.821***	0.861***
	(0.0793)	(0.0780)	(0.0782)	(0.0788)	(0.0777)
TTAX	0.178**	-0.0187	-0.0383	0.0146	-0.149
	(0.0874)	(0.0893)	(0.0900)	(0.0905)	(0.0914)
LEV		-0.190***	-0.192***	-0.179***	-0.0504
		(0.0237)	(0.0237)	(0.0265)	(0.0308)
ROE			0.120*	0.127*	-0.228***
			(0.0692)	(0.0695)	(0.0822)
SIZE				0.0937	-1.125**
				(0.501)	(0.517)
SM					0.434***
					(0.0558)
Constant	10.83***	17.99***	17.05***	14.19	33.28***
	(1.325)	(1.579)	(1.668)	(10.37)	(10.49)
Observations	1,771	1,771	1,771	1,760	1,760
R-squared	0.059	0.092	0.093	0.091	0.121
F	55.12***	59.43***	45.38***	34.93***	40.19***

Note:

***、**、*are significant at 1%, 5%, and 10% respectively; the coefficient t is in parentheses.

In Table 6, the coefficient of TS is still positive and is significant at the level of 1%, that is, income tax preferential has a significant positive impact on the investment of R&D personnel. The increase of income tax preferential will increase the investment of enterprise R&D personnel, and the income tax discount by 1%, and the average investment of enterprise R&D personnel increases by 0.861%. Therefore, the conclusion that income tax preferential has a positive impact on enterprise R&D investment has passed the robustness test. The TTAX does not pass the test, indicating that the impact of the preferential circulation tax on enterprise R&D personnel is not significant. The robustness tests also confirm the impact of SM, SIZE, and LEV on R&D investment.

The number of effective patent sales can be used as an indicator to measure the performance of R&D output [25]. The number of patents, especially invention patents, can indicate the efficiency of R&D investment [26]. A Patent is the substantive achievement of enterprise research and has a certain commercial value that can also improve the business performance of the enterprise. We replace the effect in Model 2 with the number of patent applications (patent), to use as a robustness test. The regression analysis results are shown in Table 7.

**Table 7:** Robustness tests of tax incentives on R&D output performance.

Variable	(1)	(1)	(1)	(1)	(1)
	patent	patent	patent	patent	patent
TS	0.0231*	0.0238**	0.0252**	0.0217**	0.0205**
	(0.0118)	(0.0114)	(0.0114)	(0.0104)	(0.0103)
TTAX	0.00552	0.0266**	0.0227*	0.0212*	0.0290**
	(0.0119)	(0.0120)	(0.0122)	(0.0111)	(0.0114)
LEV		0.0183***	0.0176***	0.00261	-0.00289
		(0.00291)	(0.00293)	(0.00304)	(0.00358)
ROE			0.0148*	0.0163**	0.0332***
			(0.00881)	(0.00799)	(0.00987)
SIZE				0.626***	0.672***
				(0.0601)	(0.0618)
SM					-0.0205***
					(0.00712)
Constant	2.797***	2.106***	2.020***	-10.82***	-11.52***
	(0.191)	(0.215)	(0.221)	(1.247)	(1.262)
Observations	519	519	519	517	517
R-squared	0.007	0.078	0.083	0.244	0.256
F	1.94	14.53***	11.64***	33.02***	29.29***

Note:

***、**、*are significant at 1%, 5%, and 10% respectively; the coefficient t is in parentheses.

In Table 7, it can be seen from the robustness results that after the variable replacement in the model, the coefficient symbol and significance of both the independent variables and the intermediary variables did not change significantly. The coefficient of TS is still positive and is significant at the level of 5%. Thus, income tax preference has a significant positive impact on the number of patent applications. The increase of income tax preference will increase the number of enterprise patent applications, and as the income tax preference increases by 1%, the number of enterprise patent applications increases by 2.05%. Therefore, the conclusion that income tax preference has a positive impact on the performance of enterprise innovation output has passed the robustness test.

## V. Conclusion and policy suggestion

### 5.1. Conclusion

Based on panel data of listed companies on the GEM, this paper processed and analyzed the relationship between the tax incentives and R&D input and output performance. Our research draws the following analytical conclusions. The overall level of R&D investment intensity and R&D output performance of listed enterprises on China’s GEM is high, but there are great differences between various enterprises, and the enterprise income tax preferences enjoyed by these enterprises differ greatly. Our empirical tests are consistent with Hypothesis one, and confirmed that tax preferences promote the input of R&D by high-tech enterprises. Our results are in line with McKenzie and Sershun [2], Koga [3], Czarnitzki et al. [4], and Wang [27]. From the practices of Chinese enterprises, we confirmed that through tax preference policies, the government shares the R&D risk with enterprises. This should encourage enterprises, especially high-tech enterprises, to put more investment on R&D, therefore increasing the potential for growth. The enterprise income tax discount is significantly positively correlated to the intensity of enterprise R&D investment and the intensity of enterprise human capital investment. Moreover, the income tax discount increased by 1%, the intensity of enterprise R&D investment increased by 0.369%, and the average investment of enterprise R&D personnel increased by 0.861%.

In the view of the policy’s effectiveness, tax preference, (i.e. the income tax discount) also has a significant positive impact on the performance of R&D output. Regarding the tests conducted in model 2 and the robustness tests, Hypothesis two passes. Our results are consistent with Wan et al. [13], Sun et al. [14], and Kuang and Xiao [16]. Moreover, the income tax discount increased by 1%, the performance of R&D output increased by 0.258%, and the number of enterprise patent applications increased by 2.05%.

Compared with the impact of various taxes, our tests support the hypothesis three. We found that income tax preferential treatment has a significant positive impact on the input and output of enterprise R&D. Also, the circulation tax preferential effect on the input and output of enterprise R&D is not as strong as the income tax. From session two, we noticed that the preferential income tax policies are much higher than the circulation tax. From the business perspective, the circulation tax deduction in general could only help with the cash flow of enterprises, and would not change the profit level. Therefore, the impact of circulation tax preferential treatment on R&D could be limited for businesses.

Our empirical evidence also finds the impact of firm scale. From the performance of GEM listed companies, the investment in R&D and enterprise size are negatively correlated. This indicates that SMEs are more sensitive to tax incentives, and the tax preferential policies have more impact on them. These results are also consistent with Herrera and Sanchez-Gonzalez [6] and Kobayashi [9].

### 5.2. Policy suggestions

Our conclusion based on the empirical analysis is that the tax preferential system plays a significant incentive role towards reducing the enterprise tax burden and increasing enterprise technology innovation investment intensity. Therefore, understanding how to better utilize the role of tax incentives is crucial for the long-term high-quality development of enterprises.

In order to make SMEs have greater motivation to engage in innovative activities, special incentives for SMEs should be explored. We suggest that special preferential standards must be set up for small and medium-sized enterprises. This includes clear relevant identification standards, increasing the proportion of R&D expenses, setting special tax policies by relaxing restrictions on R&D, reducing the identification standards of some high-tech enterprises, and finally, canceling the limitations of some R&D expenses.

At present, the main tax preferential method adopted in China is direct preferential. However, because the direct preferential method is applied in the later transformation stage of R&D, this is not conducive to stimulating the continuous innovation investment of enterprises. Meanwhile, indirect preferential tax functions during the early and mid-term of innovation activities which play a key role in the long-term innovation and development of an enterprise. Therefore, the government is suggested to increase the proportion of indirect preferential treatment, through tax credits on R&D. In addition, more tax-based preferential treatment programs such as accelerated depreciation and additional deductions should be adopted. By increasing the proportion of indirect preferential, the government can promote policies to effectively act on the early process of the innovation chain, and thus stimulate the innovation vitality of enterprises.

The incentive effect of preferential income tax policies on enterprises’ scientific and technological innovation ability is greater than the preferential circulation tax. This is because China’s current preferential tax policies for research and innovation of enterprises are concentrated on the enterprise income tax, there are few preferential policies involving value-added taxes. Specifically, relating to the preferential VAT policies, only the exemption for imported instruments involved in scientific research equipment and teaching, and the software developed and sold within the industry itself will be refunded. In order to make the preferential circulation tax play a greater role in encouraging enterprises to carry out scientific and technological innovation, and encourage enterprises to invest more funds into R&D activities, it is necessary to formulate more comprehensive and targeted preferential circulation tax policies for enterprises. Expanding the scope of a VAT deduction is thus conducive to reducing the direct costs of enterprises engaged in R&D activities.

In addition, high-skilled personnel is the prerequisite for enterprises to carry out R&D and innovation activities. In order to stimulate the investment and development of R&D talents, a good external environment should be created. Among them, the personal income tax policy for talented researchers should play a more positive role. Referring to the software industry, we suggest appropriately expanding the scope of individual income tax in R&D personnel to reward technical achievements. The tax requirement of the enterprise equity options obtained by R&D and technological-related managers shall be less restrictive in the range of reductions and exemptions accordingly. Meanwhile, the double taxation of individual income taxes and corporate income taxes related to dividend income could be considered to be eliminated.
